# Influencing factors on the health of nurses—a regression analysis considering individual and organizational determinants in Germany

**DOI:** 10.1186/s12913-023-09106-2

**Published:** 2023-01-31

**Authors:** Jasmin Lützerath, Hannah Bleier, Gerrit Stassen, Andrea Schaller

**Affiliations:** 1 Working Group Physical Activity-Related Prevention Research, Institute of Movement Therapy and Movement-Oriented Prevention and Rehabilitation, German Sport University Cologne, Am Sportpark Müngersdorf 6, 50933 Cologne, Germany; 2 Institute for Workplace Health Promotion, Cologne, Germany

**Keywords:** Employee health, Germany, Hospitals, Inpatient care, Nursing staff, Outpatient care, Violence, Work ability

## Abstract

**Background:**

The care sector is characterized by high absenteeism of nursing care employees due to illness. Organizational determinants that can affect the mental health of nurses are known. Although nurses are confronted with different framework conditions in different care settings, there is a lack of comparative data in Germany.

**Methods:**

The purpose of this study was to examined the relationship between work demands and employee health in different care settings. This cross-sectional survey was conducted between June and October 2021 in four acute care hospitals, seven inpatient care facilities, and five outpatient care services in Germany. 528 nursing care employees (acute care hospitals *n* = 234; inpatient care facilities *n* = 152; outpatient care services *n* = 142) participated in the survey (participation rate: 22.6%-27.9%). For each care setting, data was collected via questionnaire on individual determinants (*gender, age, profession, working time*), organizational work demands (*quantitative workload, qualitative workload, organization of work, social work climate, after work situation, verbal violence, threats, physical violence*) and employee health (*subjective health status* and *work ability*). Descriptive statistics and binary logistic regressions were performed.

**Results:**

Increasing *age* (OR = 0.650, 95% CI = 0.424—0.996) as an individual determinant and organization of work (OR = 0.595, CI = 0.362—0.978) as an organizational determinant were negatively associated with subjective health. Furthermore, age (OR = 0.555, 95% CI = 0.353—0.875), a demanding organization of work (OR = 0.520, CI = 0.315—0.858), increasing quantitative workloads (OR = 0.565, CI = 0.343—0.930) and a poorer perceived social work climate (OR = 0.610, CI = 0.376—0.991) were associated with lower work ability.

**Conclusions:**

Based on the study results, health programs should target both individual and organizational factors. The findings seem to support the importance to include nursing care employees in the planning process, as it can have an impact on their health.

**Trial registration:**

The project was registered in the German Clinical Trial Register (DRKS00024961, 09/04/2021).

## Background

In Germany, as in other countries, nursing care employees are an essential part of the health care system. Therefore, it is alarming that nursing care employees have above-average absences due to illness [1–3], especially since subjective health also influences earlier retirement [4, 5]. A further predictor of increased sick leave is self-perceived work ability [6], which is rated worse by nurses in Germany compared to other occupational groups [7].

When considering health in nursing professions in Germany, research is predominantly conducted on mental health as outcome, which is primarily assessed in the form of the latent construct burnout [8]. It is known that high perceived quantitative workloads, demanding qualitative workloads or emotionally overwhelming situations, low perceived job control, weak social support at work, and work-home interference are risk factors for the development of burnout in healthcare professionals, regardless of their setting or professional role [9]. Another important organizational aspect of work is the high incidence of workplace violence, which seems to be is common in nursing care [10–12]. It is widely accepted that experiences of violence are also associated with negative health consequences [13]. In this context, it is well known that work demands can be a predictor of mental health problems and indirectly influence the absence duration due to illness [14, 15].

Nursing care in Germany is predominantly provided in three settings: in acute care hospitals (ACH), inpatient care facilities (ICF), and outpatient care services (OCS). Basic care tasks are common to nursing employees in all three settings involving physical tasks such as the promotion and support of mobility (e.g., assistance with positioning and transfer) or assistance with activities of daily living (e.g., washing, dressing) as well as tasks of interaction such as assistance with orientation, responding to current behaviors, or the promotion of social contacts which includes dealing with the relatives [16, 17]. In addition to nursing tasks of basic care, special medically prescribed tasks are also performed that relate to diagnostics, therapy, and treatment support [18].

Beyond the tasks that are independent of the care setting, there are also varying demands.

Nursing care in ACH takes place around an acute medical event. Concurrent, the interprofessional and interdepartmental workday often requires tasks distant from patients to be organized, documented, or performed, such as patient transport, cleaning, or serving meals [19, 20]. In Germany, increasing numbers of treatment cases have been observed in recent years with a simultaneous shortening of the length of stay [21]. The resulting increased coordination of workflows requires nurses to balance medical tasks on the one hand and nursing or service tasks on the other [22].

In ICF, quantitative expectations have also increased in recent years as more requests for care placements are made [23]. Since about three quarters of the work is done with or on the residents, a much smaller part of the tasks are related to treatment care, documentation, handovers and service meetings [22]. Currently, about half of the employees (48%) in ICF are assistant staff [24], although it is anticipated that the number of assistant staff will increase and thus nurses would be given more tasks of coordination and supervision [25]. The end of a resident's stay in ICF is usually brought about by death, so accompanying those in need of care in the final phase of their lives is also a recurring task [16].

The work of OCS nursing care employees takes place in the home environment of the person in need of care, whereby individual care tasks are booked individually and partly reimbursed by the statutory insurances [26, 27]. In OCS, work is predominantly done alone in the homes of clients whose space and building conditions vary [22, 28]. Nurses spend very little time with patients, mainly in the morning, and only see them again hours or days later [22]. The work tasks also include coordination and application for services, e.g. with the general practitioner, as well as the contradiction between financial viability and the nursing care services actually needed [22, 28].

Although nursing care employees are considered a group of employees with a high health burden, analyses of possible differences in relation to care settings are still lacking [8, 9]. A better understanding of how work demands are associated to subjective health or work ability could contribute to a comprehensive setting-specific understanding and also to the development of target group specific approaches to improve the health of employees in nursing care. This has led to the following research question: *How is the association between care setting-specific work demands and employee health?*

## Methods

This cross-sectional study was conducted as part of the project "Workplace offers for health promotion and violence prevention" (BAGGer), conducted in four acute care hospitals, seven inpatient care facilities and five outpatient care services in North Rhine-Westphalia, Germany. The aim of the project was to implement and evaluate target group specific workplace health promotion for employees in nursing care. The BAGGer-project is registered in the German Clinical Trial Register (DRKS00024961, 09/04/2021). As part of the first project phase, the associations of work demands and employee health in different care settings were investigated in this study. The project phase was ethical approved by the German Sport University Cologne Ethics Committee (No. 050/2021).

### Setting & participants

Data were collected from June to October 2021. Employees of the participating care organizations were invited to take part in the survey. The inclusion criteria were 1) a minimum age of 18 years and 2) employment in a care organization participating in the BAGGer project (ACH, ICF, or OCS). Exclusion criterion was a professional activity outside of nursing in terms of patient or resident care.

### Data collection

Questionnaires were sent to all 3,484 employees in the participating care organizations with 2,200 working in nursing care (ACH *n* = 1,028; ICF *n* = 544; OCS *n* = 628) as nurses, as nursing assistants or nursing aids (e.g. in the form of a long-term nursing internship). The survey could be completed in each care organization in paper–pencil or digital format over a two-week period. Participation in the survey was voluntary and anonymous (cover sheet/ start page with information for participants about the research topic, objectives, and contact information; implied consent).

### Instrument/ measure

The survey comprised questions on sociodemographic aspects, work demands, and employee health.

The following sociodemographic information was assessed: *gender* (female, male, non-binary), *age* (18–20 years, 21–30 years, 31–40 years, 41–50 years, 51–60 years, > 60 years), *profession* (nurse, nursing assistant, nursing aid, other with free-text field) and *working time* per week (≥ 35 h, 15-34 h, < 15 h).

Work demands were assessed by care setting-specific questionnaires (BGWmiab) developed by the German Institution for Statutory Accident Insurance and Prevention in the Health and Welfare Services. The BGWmiab questionnaire contains 22 questions adapted to the specific care setting rated on a 5-point scale from "no, not at all” to "yes, exactly" [29]. The BGWmiab compromises the following dimensions: *Quantitative workload (QUANTI)* considering the amount of work in relation to time (ACH = 4, ICS = 5, OCS = 6 items), *qualitative workload* (*QUALI*) considering the emotional demands such as coping with the needs of the patients (ACH = 6, ICS = 5; OCS = 3 items), *organization of work (ORGA)* meaning the perceived job control or the opportunity to shape and influence working hours and content, including break schedules (ACH/ICS = 3; OCS = 2 items) and *social work climate (SOCIAL)* referring to the social relationships and support at the workplace (ACH/ICS = 6; OCS = 8 items) as well as *after work situation (HOME)* assessing non-work well-beeing as indicator of the degree of stress (3 items) [29]. The BGWmiab dimensions were classified according to the handbooks into “low", “under usual”, “over usual” and "high" [30–32]. These questionnaires have a sufficient to good reliability (Cronbach’s α for the five “work demands” = 0.53–0.86) and high construct validity [30–32].

Beyond, the work demands of experiencing violence on duty were assessed with the “Survey of Violence Experienced by Staff German-Version-Revised” (SOVES-G-R) [33, 34]. In the SOVES-G-R, three forms of violence are distinguished: *verbal violence* e.g. abusive or insulting language, personal verbal attacks, swearing, obscene or sexually harassing comments, *threats* e.g., warnings that you will be hurt, (sexual) harassment, physical intimidation, threats with a weapon and *physical violence* e.g., hitting, pinching, pushing, shoving, spitting, kicking, using a weapon or object as a weapon [33, 34]. For each violence forms a yes/ no question was asked to determine whether violence had been experienced by patients or family members/ visitors during the past 12 months [33, 34]. The SOVES-G-R has a good internal consistency (Cronbach's α 0.87–0.91) [35].

Employee health was determined with two variables: *subjective health* (*SH)* and *work ability* (*WA)*. In order to assess *SH*, the question "How is your health status in general?" (5-point scale from "very good" to "very poor") was taken from the Robert Koch Institute's "German Health Update" study (GEDA 2014/2015-EHIS) [36]. This question is also part of the Minimum European Health Module and has a good reliability with a correlation coefficient of 0.81 and kappa estimate of 0.74 for self-perceived health [37].

*WA* was operationalized with the Work Ability Score where the current WA is compared to the best ever WA on a scale of 0–10 [38]. The scores allow classification into: poor (0–5), moderate (6–7), good (8–9) and excellent (10) WA [39]. The single item question is showing a very strong association with the Work Ability Index [40, 41]. The Work Ability Index has satisfactory reliability (Cronbach’s α = 0.78) and a strong and consistent predictive power in a German sample [42].

### Statistical analysis

First, the data were subjected to a plausibility check by two researchers. Since the data analysis was restricted to employees working in nursing care (see  Setting & participants), the information in the free text field on the profession in particular was critically checked and, for example, persons who indicated interactive or service-providing activities in nursing, e.g. in the form of a long-term nursing internship, were classified as nursing aids.

To describe the sample, frequencies (n) and valid percentages (%) were calculated for *gender*, *age*, *profession, working time*, and experience of violence*.* Descriptive statistics (means, standard deviation (SD)) were calculated to describe BGWmiab variables of work demands and variables of employee health.

Differences in the variables between care settings were tested by Chi Square Test respectively Kruskal–Wallis-Test. To determine which setting was statistically significant different, a Dunn-Bonferroni test was performed.

To explore the association between care setting-specific work demands and employee health, two binary logistic regression models were calculated. For this purpose, dichotomized *SH* ((very) good vs. (very) poor/ moderate) and *WA* (good/ excellent vs. poor/ moderate) were used as dependent variables. Individual sociodemographic variables *gender* (female vs. male), *age* (> 40 years vs. ≤ 40 years), *profession* (nursing assistant/ aid vs. nurse) and *working time* (≤ 34 h vs. > 35 h) were included in the model as covariates. The organizational work demand variables *QUANTI, QUALI, ORGA*, *SOCIAL* and *HOME* (over usual/ high (high) vs. low/ under usual (low)) as well as *verbal violence, threat and physical violence* (yes vs. no) were included as independent variables. Care settings (*ACH, ICF, OCS*) were additionally included as dummy variables to examine the impact of these, with the setting that rated best on the employee health variables used as the reference variable.

The Hosmer–Lemeshow test was used to determine the goodness of fit of the models. The Nagelkerkes coefficient R^2^ and its effect size f^2^ was evaluated to determine the predictive power of the models. According to Cohen the effect size f^2^ = R^2^/(1- R^2^) is considered as small (f^2^ ≥ 0.02), medium (f^2^ ≥ 0.15), and large (f^2^ ≥ 0.35) in behavioral science [43]. The Odds Ratios (OR) and 95% confidence intervals (CI) were calculated.

For all analyses, the significance level was set at *p* < 0.05. Participants with missing data in dependent or independent variables were excluded from the regression models. Data were analysed using IBM SPSS 26 software (IBM Corp., Armonk, NY, USA).

## Results

### Sample description

Of 2,200 employees working in nursing care, 528 participated in the survey. This corresponds to a participation rate of 24.0% (ACH = 22.8%; ICF = 27.9%; OCS = 22.6%). The sample consisted of 440 females (84.8%) and 353 participants working as a nurse (66.9%). The largest age group were 142 employees (31.7%) between 51–60 years and 280 (54.2%) worked full-time. A more detailed sample description by care setting can be found in Table 1.

**Table 1:** Sample description by care setting

	**All care settings** *Total n* = *528*	**Acute care hospital** *Total n* = *234*	**Inpatient care facility** *Total n* = *152*	**Outpatient care service** *Total n* = *142*	**Chi**^**2**^
**Individual Variables**					
***Gender*** (*n* = 521; MD = 7)					16.00***
Female [n; valid%]	440 (84.5%)	187 (81.0%)	121 (80.1%)	132 (95.0%)	
Male [n; valid%]	79 (15.2%)	42 (18.2%)	30 (19.9%)	7 (5.0%)	
Non-binary [n; valid%]	2 (0.4%)	2 (0.9%)	**-**	-	
***Age in years*** (*n* = 521; MD = 7)					28.00***
18–20 [n; valid%]	10 (1.9%)	4 (1.7%)	4 (2.7%)	2 (1.4%)	
21–30 [n; valid%]	114 (21.9%)	65 (28.1%)	36 (24.2%)	13 (9.2%)	
31–40 [n; valid%]	114 (21.9%)	51 (22.1%)	34 (22.8%)	29 (20.6%)	
41–50 [n; valid%]	118 (22.6%)	59 (25.5%)	28 (18.8%)	31 (22%)	
51–60 [n; valid%]	142 (31.7%)	44 (19.0%)	43 (28.9%)	55 (39%)	
≥ 61 [n; valid%]	23 (4.4%)	8 (3.5%)	4 (2.7%)	11 (7.8%)	
***Profession*** (*n* = 528; MD = 0)					90.30***
Nurse [n; valid%]	353 (66.9%)	208 (88.9%)	80 (52.6%)	65 (45.8%)	
Nursing assistant [n; valid%]	76 (14.4%)	12 (5.1%)	37 (24.3%)	27 (19.0%)	
Nursing aid [n; valid%]	99 (18.8%)	14 (6.0%)	35 (23.0%)	50 (35.2%)	
***Working time*** (*n* = 517; MD = 11)					43.59***
> 35 h/ week [n; valid%]	280 (54.2%)	156 (67.5%)	77 (52.7%)	47 (33.6%)	
15–34 h/ week[n; valid%]	218 (42.2%)	73 (31.2%)	64 (43.8%)	82 (58.6%)	
< 15 [n; valid%]	19 (3.7%)	3 (1.3%)	5 (3.4%)	11 (7.9%)	
**Work demands**					
***QUANTI*** (*n* = 488; MD = 40)					
Mean (SD)^a^	2.70 (1.04)	3.05 (0.83)	2.09 (1.04)	2.66 (1.10)	64.78***
***QUALI*** (*n* = 506; MD = 22)					
Mean (SD)^a^	2.61 (1.16)	2.81 (1.25)	2.24 (1.09)	2.66 (0.99)	22.22***
***ORGA*** (*n* = 516; MD = 12)					
Mean (SD)^a^	2.94 (0.98)	2.89 (0.99)	3.13 (0.76)	2.80 (1.13)	4.98
***SOCIAL*** (*n* = 493; MD = 35)					
Mean (SD)^a^	2.30 (0.97)	2.40 (0.93)	2.20 (0.99)	2.23 (1.02)	5.70
***HOME*** (*n* = 508; MD = 20)					
Mean (SD)^a^	2.65 (1.05)	2.89 (1.15)	2.20 (0.99)	2.68 (0.73)	44.37***
***Verbal violence*** (*n* = 524; MD = 4)					
Yes [n; %]	274 (52.3%)	160 (68.4%)	70 (47.3%)	44 (31.0%)	51.49***
***Threats*** (*n* = 506; MD = 22)					
Yes [n; %]	82 (16.2%)	67 (29.8%)	10 (7.2%)	5 (3.5%)	55.55***
***Physical violence*** (*n* = 500; MD = 28)					
Yes [n; %]	137 (27.4%)	90 (39.8%)	33 (24.3%)	14 (10.1%)	38.78***
**Employee health**					
***SH*** (*n* = 520; MD = 8)					
Mean (SD)^b^	2.39 (0.69)	2.31 (0.65)	2.40 (0.71)	2.52 (0.73)	9.05*
***WA***(*n* = 516; MD = 12)					
Mean (SD)	2.36 (0.86)	2.24 (0.82)	2.57 (0.87)	2.31 (0.87)	14.65***

*MD* Missing data; range: ^a^ = [1(low)-4(high)], ^b^ = [1(very good)-5(very poor)], ^c^= [1(poor)-4(excellent)]; Chi^2^ = Differences regarding the central tendencies of the groups

^*^p < 0.05; **p ≤ 0.01; ***p ≤ 0.001

OCS employees were statistically significant older and more females than in ICF or ACH. In terms of *profession*, more nurses in ACH participated in the survey than in the other settings. In addition, *working time* varied between settings: in ACH, most full-time employed respondents were present, which was fewer in ICF and even fewer in OCS. Besides differences in individual determinants, there were also statistical significant differences in the organizational variables of work demands between the care settings. The variables *QUANTI* and *HOME* were perceived more demanding in ACH than in OCS and also more in OCS than in ICF. Further *verbal violence* and *physical violence* were experienced more often in ACH than in ICF and also more frequent in ICF than OCS. It is particularly noticeable that in ICF the *QUALI* is perceived statistical significantly lower compared to other settings and *threats* are experienced more often in ACH than in the other settings. Employee health differences between settings were found in both variables. *SH* was rated in all settings between good (2) and moderate (3), differing statistically significant in ACH and OCS. However, the situation is different for *WA*, which was rated in all settings between moderate (2) and good (3), in ICF which was statistically significantly higher than in ACH or OCS (see Table 1).

### Influencing factors on subjective health

The results of the regression analysis on *SH* can be found in Table 2. The regression analysis showed that the model on *SH* was statistically significant (Chi^2^ (14) = 28.97, *p* = 0.011, *n* = 410). *Age* over 40 years was negatively associated with a (very) good *SH* (OR = 0.650, 95% CI = 0.424—0.996). Experiencing over usual or higher demands in *ORGA* decreased the likelihood of reporting (very) good *SH* (OR = 0.595, 95% CI = 0.362—0.978). Working in *ICF* or *OCS* decreased the odds of reporting (very) good *SH* compared to working in *ACH* but this is not statistically significant. The coefficient of determination is R^2^ = 0.092 which corresponds to Cohens f^2^ = 0.10, a small effect size.

**Table 2:** Results of regression analyses on subjective health

**Variables (*n* = 410)** *R*^2^ = 0.092	**B**	**SE**	**Wald**	**Sig.**	**OR**	**95% CI**
Sociodemographic variables						
*Gender (female vs. male)*	-0.183	0.303	0.362	0.547	0.833	0.460 – 1.510
*Age (*> *40 years vs.* ≤ *40 years)*	-0.431	0.218	3.915	0.048*	0.650	0.424 – 0.996
*Profession (non nurse vs. nurse)*	-0.286	0.263	1.180	0.277	0.751	0.449 – 1.258
*Working time (*≤ *34 h vs.* > *35 h)*	-0.129	0.234	0.302	0.583	0.879	0.556 – 1.391
Work demands						
*QUANTI (high vs. low)*	-0.129	0.255	0.256	0.613	0.879	0.534 – 1.448
*QUALI (high vs. low)*	-0.348	0.232	2.255	0.133	0.706	0.449 – 1.112
*ORGA (high vs. low)*	-0.519	0.254	4.190	0.041*	0.595	0.362 – 0.978
*SOCIAL (high vs. low)*	-0.046	0.236	0.037	0.847	0.955	0.602 – 1.517
*HOME (high vs. low)*	-0.150	0.251	0.358	0.549	0.861	0.527 – 1.407
*Verbal violence (yes vs. no)*	-0.372	0.254	2.143	0.143	0.689	0.419 – 1.134
*Threats (yes vs. no)*	-0.005	0.325	0.000	0.988	0.995	0.526 – 1.883
*Physical violence (yes vs. no)*	0.053	0.276	0.037	0.847	1.055	0.614 – 1.812
Care setting variables						
*ICF vs. ACH*	-0.525	0.325	2.604	0.107	0.579	0.313 – 1.119
*OCS vs. ACH*	-0.547	0.297	3.381	0.066	0.591	0.323 – 1.037

R^2^ = Nagelkerkes coefficient of determination; B = regression coefficient; *SE* Standard error, *Sig*. Significance, *CI* Confidence interval

^*^p < 0.05; **p ≤ 0.01; ***p ≤ 0.001

### Influencing factors on work ability

The results of the regression analysis on *WA* can be found in Table 3. The second regression also showed that the model (Chi^2^ (14) = 72.00, *p* < 0.001, *n* = 405) was statistically significant. There was a statistical significant negative association of increasing *age* and “good/ excellent” *WA* (OR = 0.555, 95% CI = 0.353—0.875). For the work demands variables, the odds of reporting “good/ excellent” *WA* decreased when *QUANTI* (OR = 0.565, 95% CI = 0.343—0.930)*, ORGA* (OR = 0.520, 95% CI = 0.315—0.858) and *SOCIAL* (OR = 0.610, 95% CI = 0.376 – 0.991) were perceived as over usual or high demands. The odds of reporting “good/ excellent” *WA* were lower in *ACH* and *OCS* than in *ICF*, however this is not statistically significant. The coefficient of determinants is R^2^ = 0.218 and Cohen f^2^ = 0.28, which correspondents to a medium effect size.

**Table 3:** Results of regression analyses on work ability

**Variables (*n* = 405)** R^2^ = 0.218	**B**	**SE**	**Wald**	**Sig.**	**OR**	**95% CI**
Sociodemographic Variables						
*Gender (female vs. male)*	-0.259	0.328	0.648	0.430	0.772	0.406 – 1.468
*Age (*> *40 years vs.* ≤ *40 years)*	-0.588	0.232	6.426	0.011*	0.555	0.353 – 0.875
*Profession (non nurse vs. nurse)*	-0.180	0.280	0.414	0.520	0.835	0.483 – 1.445
*Working time (*≤ *34 h vs.* > *35 h)*	0.030	0.247	0.015	0.902	1.031	0.635 – 1.672
Work demands						
*QUANTI (high vs. low)*	-0.571	0.255	5.037	0.025*	0.565	0.343 – 0.930
*QUALI (high vs. low)*	-0.443	0.241	3.388	0.066	0.642	0.401 – 1.029
*ORGA (high vs. low)*	-0.654	0.256	6.550	0.010**	0.520	0.315 – 0.858
*SOCIAL (high vs. low)*	-0.494	0.248	3.979	0.046*	0.610	0.376 – 0.991
*HOME (high vs. low)*	-0.327	0.253	1.666	0.197	0.721	0.439 – 1.185
*Verbal violence (yes vs. no)*	-0.135	0.262	0.264	0.607	0.874	0.523 – 1.461
*Threats (yes vs. no)*	-0.276	0.354	0.610	0.435	0.759	0.379 – 1.518
*Physical violence (yes vs. no)*	-0.455	0.292	2.420	0.120	0.635	0.358 – 1.125
Care Setting Variables						
*ACH vs. ICF*	-0.406	0.334	1.479	0.224	0.666	0.346 – 1.282
*OCS vs. ICF*	-0.458	0.332	1.895	0.169	0.950	0.330 – 1.214

R^2^ = Nagelkerkes coefficient of determination; B = regression coefficient; *SE *Standard error, *Sig.* Significance, *CI* Confidence interval

^*^p < 0.05; **p ≤ 0.01; ***p ≤ 0.001

## Discussion

To our knowledge, this study is one of the first to focus on both the associations of individual and setting-specific organizational factors on employee health in the nursing settings ACH, ICF, and OCS in Germany. Main finding of the presented study was that increasing *age* and higher work demands in *organization of work* showed a negative association with both variables of employee health. Furthermore, on an organizational level, higher demands in *quantitative workload* and a demanding *social work climate* were negatively related to *work ability*.

### Considering the individual determinants

German sick leave data on nursing care employees indicate that sickness rates increase with age, being female, assistant staff or part-time employed [2, 3, 44]. In this study, only *age* was identified as an influencing factor on employee health variables predicting sick leave.

First, sociodemographic data are discussed to determine if the study population differs from the German nursing population. While in the presented study 58.7% of all nursing employees have the *age* > 40 years, in Germany this *age* group is represented by 62.0% [45]. Moreover, in Germany 83% of the nursing workforce is female, here it is 84.5% [24]. In regard to *profession*, the proportion of nursing assistants and nursing aids in Germany is nearly the same in the presented study for ACH 11% (here 11.1%) and ICF 47% (here 47.3%) but lower in OCS 39% (here 44.2%) [24]. It is also known that nursing employees are more likely to work part-time with increasing age [2]. While the respondents in this study are younger than in Germany this is also reflected in the *working time* per week. In Germany, 57.6% of the nursing care employees work part-time [45], whereas in this sample it is 45.8%. This could support the findings that health is more influenced by *age* than *working time*.

Although *work ability* is a predictor of sick leave, studies also showed the contrary effect that being female *gender* is positively associated with *work ability* among nursing assistants [46]. Other authors, on the other hand, conclude that lower *professional* status leads to more physical complaints [47]. However, it also appears that *age* and *gender* have no influence on emotional exhaustion in employees with the *profession* nurse [48]. If only the *subjective health* is considered, females in Germany rate it worse than males, but the difference is only statistically significant in the age group of 18 to 29 year-olds [49], which could be one reason why *gender* does not appear as an influencing factor in this study. Consistent with the findings of this study, younger *age* is also associated with a higher *work ability* [46, 50]. Although no association between older *age* and physical *work ability* can be found when nurses have low levels of burnout symptoms [50]. Therefore, it seems that in addition to physical fitness, personal coping with stress is also important when it comes to employee health as we age.

### Considering the organizational determinants

All included work demands are known to be associated with burnout [9, 13]. However, in the present study only *organization of work, quantitative workload*, and *social work climate* showed an statistical significant effect on employee health variables. In the existing international literature, resources in autonomy, i.e. low demands in *organization of work*, demands in *quantitative workload* and resources in various aspects of *social work climate* are also identified as key issues in health-related outcomes [51]. This is supported by the findings of nurses in Germany, which show that low decision latitude in the *organization of work,* high *quantitative workload*, and a weak percieved *social work climate* are key stressors associated with emotional exhaustion [48].

*Organization of work* is perceived as particularly demanding when processes are determined by others. One aspect that seems to be responsible for this percieved low job control is the frequent filling in for colleagues, leading to more (weekend) working hours [52, 53]. The burdens of compliance with working time regulations have been further exacerbated by the pandemic in Germany [54]. In this context, it is known that overcommitment, e.g. when filling in for colleagues, and working more than two weekends per month are negatively associated with *subjective health* in nursing care employees [55]. Moreover, demands in *organization of work* are characterized by work and break interruptions, which requires a high level of multitasking [53]. In addition, both work and break interruptions are often associated with increasing *quantitative workload* [56, 57]. Occupational health research in Germany has found that nurses who experience frequent work interruptions and multitasking not only show signs of mental exhaustion, but they also rate their overall *work ability* lower [57]. It is known, especially among older employees, that the negative stress consequences decrease with additional short rest breaks [58, 59]. Short breaks not only have the potential to reduce stress but also to promote social interaction [60]. This seems to be desirable, as negative effects on employee health due to *quantitative workload* are buffered when the *social work climate* is experienced as good [61].

Unlike research on mental health as an outcome, *qualitative workload, after work situation* and *violence* showed no statistical significant association with employee health variables. This could be related to the fact that *qualitative workload* is perceived as demanding by caregivers especially when exposed to it alone without peer support [53]. Especially since the beginning of the pandemic, social support and trust among colleagues and with superiors have become even more important for nursing care employees in Germany [52]. In this context it is known that a high level of social support is capable of being a mediator of mental strain even since the outbreak of the pandemic [62]. In this study sample, demands in *social work climate* were rated as under usual in all care settings. Therefore, it could be that the positively rated *social work climate* buffered the effects of *qualitative workload* in this study sample. The questions on *after work situation* are indicators of the degree of stress [29]. Although *after work situation* was found over all settings as over usual demanding no statistical significant association was found. Among other things, this could be due to the fact that German nurses who find it problematic to switch off after work, or even experience mental, physical and social burdens, nevertheless seem to consider their health as good [53].

Furthermore, *violence* was experienced far less frequently in this study than in Germany, which could be one reason why no statistical significant association was found. Within one year, almost every nurse in Germany experiences *verbal violence* (84–97%), 21–55% experience *threats*, and 61–77% experience *physical violence* [11, 12]. In comparison, 52.3% experienced *verbal violence*, 16.2% *threats*, and 27.4% *physical violence* in this study. One reason for this could be that caregivers do not perceive assaults as violence if they associate them with the illness of the person in need of care, even if they are experienced on a daily basis [53]. It is known that nurses who have received training on violence are more likely to perceive assaults as violence, thus increasing the number of incidents [63]. Therefore, it might be beneficial to further raise awareness of this issue among nursing care employees.

### Considering the care setting

It is known that absenteeism due to illness varies between care settings in Germany: ICF has the highest followed by OCS and in ACH these are the lowest [1]. In contrast, the present study has shown that the care setting made no statistically significant contribution to employee health. Supporting this, no statistical significant differences in *subjective health* between settings are found in Germany [64]. In contrast, statistical significant differences in physical health were found between employees of ACH and ICF or OCS in Germany [65]. Other authors further describe differences in *work ability* between German care settings [66]. However, it should be noted that the data in those studies are purely descriptive and not adjusted to individual or organizational determinants.

Therefore, it seems that individual and organizational factors are of considerable importance for the employee health of nursing staff in Germany, regardless of the care setting.

### Limitations

The aim of the study was to find approaches for health promotion programs in nursing. Thus, a strength of this study is that employee health was not limited to mental health. In addition, the data were generated in different nursing settings, which means that the statements on the influencing factors relate to a broader target group of nursing staff.

However, the results of this study must be considered with some limitations.

One issue is that the data were collected in organizations participating in a health promotion project which could indicate a possible selection bias. In the participating organizations, it is particularly in ICF and OCS that there is a high level of health promoting capactiy, which depends on health promoting willingness and structured health promoting management [67]. Therefore, it could be assumed that employees in these organizations experience fewer work demands or that these are already being addressed in a preventive manner.

Furthermore, the response rate of the 2,200 employees working in nursing care was only 24%. It remains unclear whether individuals with lower or higher employee health participated in the survey. In terms of individual determinants, it is also not known whether the respondents have private care duties or financial responsibilities. Although it was known that work-family conflicts and lack of formal rewards could have an impact on employee health [9, 51], they were not fully captured by the chosen instrument. This could be considered in future studies.

In the data analysis, the variables were dichotomized and employee health was accessed with single item questions, which can lead to bias. The regression models in this study showed a small effect size for *SH* and a medium effect size for *WA*, so the results should be interpreted with some caution.

## Conclusions

This study has contributed to the identification of key work demands in order to develop target group specific approaches to improve the health of employees in the care sector. It seems that different approaches in different care settings are not required for the development of health promotion programs in nursing but rather approaches that target individual and organizational determinants.

From an individual perspective, it seems particularly important to pursue approaches that address the needs of aging employees. This could include approaches to promote physical fitness as well as stress management.

From an organizational perspective, approaches could target the reasons why *organization of work, quantitative workload*, and *social work climate* are perceived as demanding. Therefore, involving (older) nursing care employees in the process of redesigning work structures, processes and times could be benificial, as this could give them more job control. Redesigning could include, for example, creating a work environment that allows for social gathreing and breaks. This could be supported by a higher staffing ratio and reducing non-patient related tasks. In addition, social events and communication training could strengthen the social interaction.

Nevertheless, more data on health promotion programs for nursing care employees are needed to identify which interventions are being implemented, how, and for whom in each setting, and whether they are able to reduce work demands and thus improve employee health.

## Data Availability

The data presented in this study are available on request from the corresponding author.
